# “It's like a swan, all nice and serene on top, and paddling like hell underneath”: community first responders’ practices in attending patients and contributions to rapid emergency response in rural England, United Kingdom—a qualitative interview study

**DOI:** 10.1186/s13049-023-01071-3

**Published:** 2023-02-13

**Authors:** Gupteswar Patel, Viet-Hai Phung, Ian Trueman, Julie Pattinson, Vanessa Botan, Seyed Mehrshad Parvin Hosseini, Roderick Ørner, Zahid Asghar, Murray D. Smith, Elise Rowan, Robert Spaight, Jason Evans, Amanda Brewster, Pauline Mountain, Aloysius Niroshan Siriwardena

**Affiliations:** 1 Community and Health Research Unit, School of Health and Social Care, University of Lincoln, Lincolnshire, UK; 2 East Midlands Ambulance Service NHS Trust, Nottingham, UK; 3 National Ambulance Commissioners Network, London, UK

**Keywords:** Community first responders, Practice wisdom, Emergency care, United Kingdom

## Abstract

**Background:**

Community First Responder (CFR) schemes are a long-established service supplementing ambulance trusts in their local community in the United Kingdom. CFRs are community members who volunteer to respond to people with life-threatening conditions. Previous studies highlighted the motivations for becoming CFRs, their training, community (un)awareness and implications of their work on themselves and others. The practices of CFRs in prehospital care remain underexplored. Therefore, we aimed to explore real-world practice of Community First Responders and their contribution to prehospital emergency care.

**Methods:**

We conducted 47 interviews with CFRs (21), CFR leads (15), ambulance clinicians (4), commissioners (2) and patients and relatives (5) from six ambulance services and regions of England, United Kingdom. Thematic analysis enabled identification of themes and subthemes, with subsequent interpretation built on the theory of practice wisdom.

**Results:**

Our analysis revealed the embeddedness of the concept of doing the right thing at the right time in CFR practice. CFRs’ work consisted of a series of sequential and interconnected activities which included: *identifying* patients’ signs, symptoms and problems; *information sharing* with the ambulance control room on the patient’s condition; providing a *rapid emergency response* including assessment and care; and *engaging* with ambulance clinicians for patient transfer. The patient care sequence began with recognising patients’ signs and symptoms, and validation of patient information provided by the ambulance control room. The CFRs shared patient information with ambulance control who in turn notified the ambulance crew en-route. The practices of CFRs also included delivery of emergency care before ambulance clinicians arrived. Following the delivery of a rapid emergency response, CFRs engaged with the ambulance crew to facilitate patient transfer to the nearest medical facility.

**Conclusion:**

The sequential CFR practices supported ambulance services in delivering prehospital and emergency care in rural areas. CFR practices were founded on the principle of practice wisdom where CFRs constructed their practice decisions based on the patient’s condition, their training, availability of equipment and medications and their scope of practice.

**Supplementary Information:**

The online version contains supplementary material available at 10.1186/s13049-023-01071-3.

## Background

The National Health Service (NHS) England has prioritised community engagement in decision-making and support for future healthcare services [1]. Community First Responder (CFR) programmes have been introduced and implemented throughout England to achieve the objective of community engagement in providing a rapid emergency response, particularly in rural communities where longer ambulance wait times are more common. CFRs are lay members of the general population who have received varying levels of prehospital emergency care training, or off-duty health professionals, who volunteer to offer clinical support to Emergency Medical (ambulance) Services (EMS) in delivering emergency care to people suffering from life-threatening conditions, such as myocardial infarction, stroke or cardiac arrest, while an ambulance is en-route [2, 3]. Provision of EMS varies based on the patient’s geographical location, with waiting times for medical assessment likely to be higher in semirural and rural regions than in urban areas [3, 4].

Our own research has identified that the roles of CFRs are important in EMS delivery and augment the overall service provision by rural ambulance services [5]. CFRs thus play an important role in the rural health and care workforce, where timely access to emergency care can be more challenging. The CFR workforce does not replace ambulance clinicians; rather, they complement the ambulance service response [2, 3, 5].

Available literature on CFRs have primarily focused on understanding their motivation to become CFRs [3, 6–8], implications of CFR roles on mental wellbeing [9], CFRs’ role in improving access to defibrillation [10], and operational strategies and challenges [6]. For example, Barry et al. [11] identified various strategies of CFR implementation and associated challenges: challenges included problems of group or interprofessional dynamics and conflicting opinions on CFRs’ scope of practice. The authors discussed strategies for implementation, which included periodic recruitment, screening and selection, and mechanisms for supportive induction and training. Another large body of CFR literature has recognised the critical role of CFRs in rural areas [12–15]. However, the real-world practices of CFRs and their role in prehospital care have remained underexplored. Hence, this study goes beyond technical assessment of CFRs’ contribution and seeks to explore the practices of CFRs while attending patients.

### Conceptual framework

The concept of practice has been increasingly adopted in organisational, healthcare and social science scholarship, and a remarkable growth in analysis using terms such as practice, interaction, activity and performance have been observed [16]. A practice-oriented analysis seeks to emphasise learning about real-world practices, which involves practitioners’ actions in particular circumstances. Thus, practice represents the rational behaviours of individuals and groups of individuals with similar interests within organisational and social processes [16]. Moreover, Schatzki [17] argues that interests in practice theory are based on the phenomena of “knowledge, meaning, human activity, power of science and language”.

Practical wisdom is analogous to competent judgement—doing the right thing at the right time and for the right cause or reason. It entails the capacity to observe and understand a situation’s complexities, empathise suitably, and act effectively in the real world [18]. It is distinct from scientific knowledge in that it encompasses all dimensions of social lives, including truthfulness, integrity, and open-mindedness. Additionally, practical wisdom reflects the goal of striking a balance in action in order to rein in extreme decision-making until additional support emerges, and it is inextricably related to a diverse range of everyday activities [19].

## Methods

### Aim of this investigation

We aimed to explore the practices of CFRs while attending patients and delivering care in six ambulance service regions in England. The exploration was grounded on the research questions: How and what are the practices CFRs perform while attending patients in the community?

### Study design and setting

This qualitative research was undertaken in six ambulance service regions in England, United Kingdom. The six research sites were rural ambulance services (see Table 1), which enabled us to understand the role of CFRs in rural healthcare, where their services are crucial. This study presents a subset of the findings from a larger study undertaken at the University of Lincoln, United Kingdom.

**Table 1:** Participants across the ambulance services

Ambulance services	CFRs	CFR leads	Ambulance clinicians	Commissioners	Patients and relatives
EMAS	6	2	0	1	1
SCAS	3	3	0	0	2
SECAMB	3	7	1	0	0
SWASFT	0	1	0	0	0
WMAS	3	2	3	1	0
YAS	6	0	0	0	2
Total (47)	21	15	4	2	5

*CFR* Community First Responder, *EMAS*: East Midland Ambulance Service NHS Trust, *SCAS* South Central Ambulance Service NHS Foundation Trust, *SECAMB* Southeast Coast Ambulance Service NHS Foundation Trust, *SWASFT* South West Ambulance Service NHS Foundation Trust, *WMAS* West Midland Ambulance Service NHS Foundation Trust, *YAS* Yorkshire Ambulance Service NHS Trust

### Data collection and participants

We obtained the contact information of various stakeholders who were actively involved in the CFR schemes at the time of data collection, from the respective ambulance services, and based on the roles and responsibilities, years of experience and locations, we sent participant information statements and consent forms to invite them to participate in the study giving them time to make an informed decision. Participants who provided us with written consent were recruited. Thus, we ensured voluntary nature of participation. We interviewed 47 different stakeholders involved in the functioning of CFR schemes to explore and understand how CFRs actually practice when attending patients (see Table 1). Purposive sampling [20] was employed in this study to include a diverse range of stakeholders engaged in the implementation of CFR schemes and to collect extensive information [21] on CFR practices until data saturation was achieved. In-depth semi-structured interviews are the most widely used data collection approach in qualitative research [22, 23], as they encourage the participant to discuss in detail the topic under exploration. Interviews were conducted by three researchers (VHP, JP and IT), guided by interview schedules, and separate interview schedules were developed based on participants’ roles and responsibilities in order to incorporate a wide range of experiences and practices of the different stakeholders. The data collection was conducted from April 2020 to December 2021, at the peak of the COVID-19 pandemic and periodic nationwide lockdown, which led us to conduct interviews online [24, 25]. The interviews were audio-recorded with the participants’ written consent and subsequently transcribed. The duration of the interviews ranged from 36 to 94 min.

For this study, a patient and public involvement (PPI) panel was established [26], and PPI members were debriefed about the emergent data and study findings in order to obtain data validation and reflections for further exploration.

### Data analysis

A thematic analysis approach [27] guided data analysis, and the subsequent interpretation was informed by the theoretical concept of practice wisdom. In this study, the thematic analysis comprised of several steps in which we organised and synthesised data by producing shortcodes, grouping the shortcodes, and synthesising meaning based on broader emergent themes. Four researchers (GP, VHP, IT and JP) inductively coded transcripts using NVivo12 and constructed a comprehensive list of codes as they emerged from the data. Cross-reading and reflexivity were performed periodically within the research team, and the codebooks were combined to create a coding framework. The coding framework was then amended following a series of discussions among research team members (GP, VHP, JP, IT and NS). Nvivo12 supported the production of data outputs for each code. The data outputs were read, reviewed and re-read to identify higher-level analytical themes. At this stage, the practices of CFRs and the entrenched concept of practice wisdom emerged as a crucial theme for understanding the sequence of practices performed by CFRs to deliver emergency care. A 32-item checklist based on the Consolidated criteria for reporting qualitative research (COREQ) has been used to establish and maintain standardised reporting in qualitative research. (see Additional file 1).

## Results

The intention and practice of CFRs to attend patients in emergency conditions were an attempt to stabilise the patient’s condition and to bridge the time gap to arrival of ambulance clinicians, in line with the notion of “doing right thing at the right time” [17, 28] in the practical wisdom theory. The CFR roles and practices to respond to patients in emergencies were consistent with their broader goal and motivation of serving the community, and highlighted CFRs’ decision making as well as practical wisdom in their efforts to discover the appropriate decision in each situation. Analysis of the CFR practices revealed a series of sequential and interconnected activities, such as *identifying* patients’ signs, symptoms and problems; *information sharing* with the ambulance control who would share this with the ambulance clinicians on the patient’s condition; providing a *rapid emergency response* including assessment and care; and *engaging* with ambulance clinicians on arrival. Although these CFRs' practices, from identifying signs and symptoms to engaging with ambulance clinicians, were not the same for all cases, the most salient themes emerging from the data are presented below.

### Identification of patients’ signs and symptoms

When arriving at the scene, CFRs attempted to verify information provided by the ambulance control centre by compiling and comparing it with their own observations as well as that from patients and family members, while assessing possible risks by paying attention to various signs and symptoms. Following this process of information validation, the CFRs constructed their initial impression of the patient’s condition by assessing the patient's body posture, breathing pattern, suspected bleeding, and pulse rate. They also employed a variety of instruments and diagnostic procedures to validate their assessment and the severity of the patient's condition. In addition, CFRs noted their initial impression and filled out a clinical record, the Patient Record Form (PRF), which was in electronic form in some regions.

Often the patient information received from the control room did not match with CFRs’ first-hand observations recorded, as one CFR noted:

It immensely varies on what sort of cases I attend. A lot of times things [patient information] will come through and it [control room] will say unconscious, not breathing or whatever, and I walk in the room and they're [patients] sat at the kitchen table conscious and breathing. But in some cases, patients’ conditions are severe than what they [control room] said. (WMAS_CFR_300921)

The quote above illustrates the discrepancy between what CFRs were briefed in relation to the patients’ condition and what they encountered on scene. Hence, CFRs were unable to rely on the preliminary information and often needed to assess a patient’s condition on arrival. In most instances CFRs expressed this mismatch in information provided that assessed the condition as being serious, and prompted them to inform the ambulance control about the actual patient condition. This information was also shared with the ambulance clinicians en route to alert them to the actual situation or patient condition, discussed in the next section.

### Information sharing

An explicit information structure was apparent, and reflected in the process of information gathering and sharing by CFRs. Information sharing practices required time and effort during a critical period for the patient, with information imparted through informal telephone conversations or formal documentation. The formal approaches were automated and standardised in some regions where the CFRs were required to use an electronic patient record form (e-PRF). CFRs documented the patient’s condition and key measurements on the e-PRF which was often automatically electronically shared with the ambulance crew and the ambulance service control room. Elsewhere, paper-based PRFs were used to document and share information with the ambulance crew while performing handover, as demonstrated in the following excerpt:

Most of us have paper PRFs, whereas the [anonymised-ambulance service] CFRs have tablets. So when we are on-scene, observing blood pressure, temperature, respiration rates, oxygen saturation, we document them on our paper PRF, and hand that over to ambulance crew when they arrive. (SCAS_CFR_181021)

We found the information-sharing practices of CFRs emphasised a collaborative relationship between ambulance services and CFRs. Timely information-sharing practices were performed as a critical step in providing care. CFRs were the first contact point between the patients and emergency care providers within the community, and precise information gathered was seen as important for the patient care process. These information-sharing practices helped ambulance staff to anticipate and recognise the severity of the patient illness or signs of patient deterioration and enabled them to plan their care strategies and triage treatment decision-making. These early assessment and care strategies were also helpful in minimising the time for appropriate medical intervention.

Once we reach, we do a proper assessment and take observations, patient history. if they [patient] are sick enough, we get back in touch with the desk [control room], and tell them the severity of the patient, we need help quicker, when is help coming? We also ring up to the clinical team if we need more help and support from what we have found. (EMAS_CFR_210921)

Similarly an ambulance clinician noted:

When we respond to emergency calls, generally we see a CFR already on scene and have started patient assessment and sometimes treatment too. We listen to what they have to say first before handover. (SECAMB_Ambulance _111021)

The above excerpt emphasises the significance of CFRs’ practices of information-sharing with the ambulance control as well as the ambulance clinicians. These practices also aligned with the concept of practice wisdom [18], which refers practitioners to do the “right thing at the right time” (p03). The embeddedness of practice wisdom was exemplified in the CFRs’ practices of expediting ambulance arrival on-scene when the intensity of the patient condition required this, particularly in rural areas with poor roadways.

They do. If they have valuable information to give us, this is what's going on, this is the location you need to go to. (SECAMB_Ambulance_111021)

The information sharing practices of the CFRs were valued by the ambulance clinicians in optimising the pathways of providing medical care to patients in emergencies: *“Some crews really value the input of the CFR and the information… So communication is really good and important.”* (YAS_CFR_ 180,721)

Information gathering and sharing were crucial and led to the decision-making for delivering appropriate and timely emergency care within the communities, which we will discuss in the next section.

### Rapid emergency response

Rapid emergency response practices followed and were informed by the information sharing step. Emergency responses provided by CFRs were dependent on the patient’s condition and their scope of practice. CFRs often attended patients with breathing difficulties, chest pain, acute coronary syndrome, stroke, epilepsy, cardiac arrest, burns, trauma, asthma, diabetes, unconsciousness, and falls. The most commonly reported forms of rapid emergency responses included: oxygen administration; defibrillation; clearing airways obstruction; cardiopulmonary resuscitation (CPR); and lifting older patients who had fallen.

For example, the process of oxygen administration was informed through oxygen saturation measurement, which CFRs conducted in the identification of signs and symptoms step. CFRs attending patients with lethargy or restlessness or difficulties in breathing, on measuring oxygen saturations identified the requirement for immediate oxygen administration, which they were equipped with and which was within their scope of practice.

If there's anything we can do for them in terms of what we carry, we do the best possible. for example if somebody has got difficulty breathing, and need oxygen or salbutamol, then we administer the oxygen and give them salbutamol. (SECAMB_CFR_ 111,021)

A patient noted:

I’ve never really thought is it something that happens before an ambulance. But a CFR came last time, administered oxygen and that was a quick relief. (YAS_Patients_120521)

Patients with cardiac disorders were another frequent category of patients attended by CFRs. CFRs performed CPR and defibrillation and responded to patients with heart failure, acute coronary syndrome and myocardial infarction by assessing their condition. In a range of instances, these rapid measures were crucial for stabilising the patients’ condition in the community, while waiting for the ambulance. Although CFRs performed CPR and defibrillation, they expected the ambulance to arrive quickly considering the patient’s condition, which was often critical or deteriorating, in the face of CFRs’ more limited skills, resources and scope of practice.

It depends on what it is really. If it is a cardiac arrest, then we will just start CPR and defibrillation immediately, and we will get on with that and when they [ambulance crew] turn up, they will take over. If we suspected it was a heart attack, there's nothing we can do. We don't have anything that will stop that happening, but what we can do is keep them calm, put them in a good position and do some observations. If it's a stroke, there's nothing we can do to make any difference, except again to keep them calm and do observations, monitor it and report back to the desk if things are deteriorating; which we do. (EMAS_CFR_270921)

In contrast, the CFR response was more limited in cases where the interventions required were beyond the scope of CFR practice, and where CFRs were obliged to follow the guidelines set out by the ambulance service which they were affiliated to. Our findings suggest the types of patients which CFRs were less likely to attend were maternity cases (particularly during labour), road traffic collisions, fire emergencies, patients with mental health disorders, and children. The practice of not delivering an emergency response in such cases was aligned with the practical wisdom of waiting for additional support before making *harsh* or difficult decisions. These CFR practices were important for patient safety and to ensure legitimate boundaries of practice for CFRs.

We basically just be there to monitor the patient until the crew comes; once they arrive we handover the observation, help the crew if they need, else we leave if they don’t need us. (SECAMB_CFR_111021)

In summary, emergency response practices of CFRs contributed to the overall purpose of stabilising the patients’ condition before the arrival of the ambulance. In addition to these technical contributions, the CFRs frequently interacted with patients and their family in order to (re)assure them that clinical attention was on its way. Skills in patient communication were not substantially a part of their training and these skills were personal to individual CFRs. The CFRs’ communication comprised patient information in connection with their current state, information about early interventions they could provide and maintaining optimism in relation to treatments and outcomes.

I think communication with people is the biggest part of our role. It's like a swan, all nice and serene on top, and paddling like hell underneath. Sometimes you can’t do a lot but you have to reassure them [patients and relatives]. The biggest skills is communication. I say this to any new CFRs. there are two sides that you have to develop as a CFR, one is the communication side, and the others. (EMAS_CFR_200921)

The above excerpt suggests that the rapid emergency response of CFRs included not only technical measures but also a substantial communication component, which was linked with the broader principle of CFR schemes stabilising patients in the community before EMS arrival.

The decision-making in relation to the emergency response was made by CFRs in line with the availability of on-scene treatment or services. The decision making was dependent on the type of case, context and speed or timing of arrival. For example, in instances when CFRs and ambulance arrived together, CFRs were less likely to conduct assessments and ambulance clinicians eventually took over care.

### Engagement with ambulance clinicians

Before the ambulance arrived, CFRs delivered patient care as noted above in the rapid emergency response section, and then handed over to ambulance clinicians, followed by completing a structured PRF, either on paper or digital format. CFRs recognised the arrival of ambulance clinicians and handover as final part of their direct role in patient care and approached the ambulance clinicians to support them in any measures required to facilitate patient transfer. Often ambulance clinicians sought assistance from the CFRs in the patient transfer, however, in some instances, CFRs were asked to leave.

I was staying and help and because I am on duty. Sometimes I help them to take the stretcher out, prepare other ambulance process to load the patient. They [ambulance crew] also appreciate it. (YAS_CFR _180721)

The engagement of CFRs with the ambulance crews comprised of several activities, including preparing the ambulance to carry the patient, repositioning the ambulance vehicle, and using a riser for falls patients.

They [ambulance crew] say – Here’s the keys can you go and put down the rear doors. Here are the keys, can you drive further up or reverse in. (EMAS_CFR _130921)

A couple of times in the past I've been asked to be a third man on the back of an ambulance to help transporting patients to hospital. (WMAS_CFR _290721)

I attended a cardiac arrest patient. A CFR was already there, doing CPR and giving oxygen. That was a busy day, and no other ambulance and support available. So we used CFR as our second resource, and it turned out to work. The patient was in bad shape, and the CFR knew what to do. (WMAS_Ambulance_070921)

The above excerpts illustrate how CFRs engaged and supported the ambulance crews within the community. Often, these CFR practices exceeded their obligations, as the CFR policies usually limited the responsibilities of CFRs. The embeddedness of practice wisdom by doing the right thing at the right time was evident in the CFRs’ engagement with ambulance clinicians by moving beyond the policy obligations of CFRs. CFRs’ engagement facilitated crews’ efforts lifting and transferring patients to the ambulance, enhancing conveyance to hospital, timely intervention and patient survival.

## Discussion

**Fig. 1:**
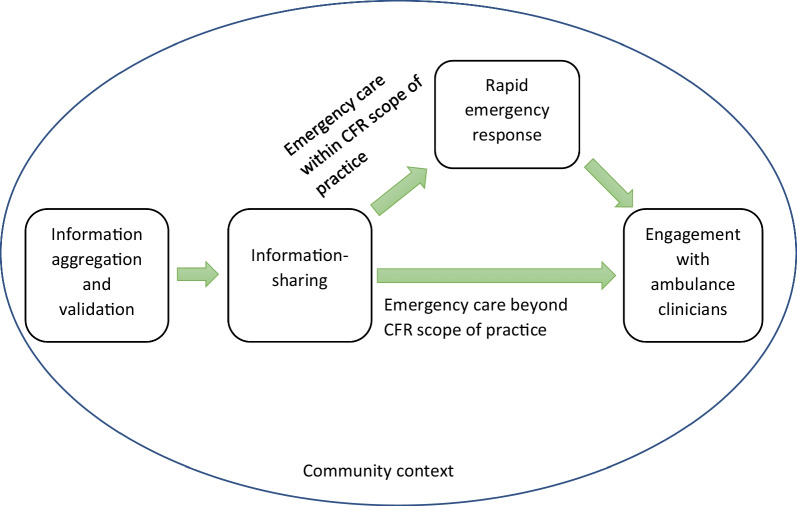
Conceptual map of community first responder practices

This study explored the varied practices performed by CFRs in England’s six ambulance service regions while responding to patients during emergencies. A series of sequential and interrelated actions of CFRs have emerged as crucial in bridging the time gap between the arrival of ambulance clinicians and access to prehospital care, hence contributing to early assessment and intervention or treatment. Receiving an alert from the ambulance control and initial patient information paved the way for the next stage of CFRs reaching patients. The response of the CFRs to patients started with the acquisition of further patient information and validation of data obtained from the control room. CFRs exchanged complete and precise patient information with ambulance control for triaging purposes and to update responding ambulance clinicians so they were more prepared on arrival. CFRs provided a rapid emergency response, including CPR and defibrillation. The final step was to engage with ambulance crews to expedite patient transfer and minimise transfer time. Thus, CFR practices were vital in emergency healthcare situations, especially for patients with life threatening conditions, by delivering direct or indirect care interventions. These practices were especially important when ambulance arrival was delayed (Fig. 1).

Although CFR practices appeared to be important in providing prehospital emergency care, the issues inherent in decision-making were ingrained in these activities. These dilemmas corresponded strongly with the theory of practical wisdom of “doing the right thing at the right time” [18]. The set of CFR practices was guided and defined by CFR policies within which they performed, without breaching their legitimate scope of practice. Individual CFR actions were often unique and spontaneous, but they were driven by the context or patient condition, as well as the structure or policy of the CFR schemes within which they functioned. These phenomena are explained by critical realism theory [29], which states that structure and culture shape the situations or contexts that individuals confront and determine their creative path of action to deal with situational constraints. In certain instances, CFRs were unable to deliver emergency care and had to wait for the ambulance to arrive. These occurrences were also consistent with the practical wisdom, in which the service provider was required to await the arrival of more assistance before making difficult decisions [30].

This study is the first of its kind in analysing how and what precisely CFRs do while attending patients in emergencies in the community. We highlight the contribution of CFRs to the overall emergency care delivery process, as Barry et al. (2018) also found with CFRs significantly adding human resources and value to the statutory ambulance service response in managing out of hospital cardiac arrest survival in Ireland and facilitating the meeting of organisational goals by ambulance services. Similarly, we found that CFRs practices supported the concept of “chain of survival” [31] from cardiac arrest, and that the CFRs strengthened access to early assessment and early intervention [10]. Furthermore, this study goes beyond Barry et al.’s (2019) analysis of the CFRs’ role in out of hospital cardiac arrest care with basic life support, care for the families, assisting ambulance staff and dilemmas in care decision making, and highlighted a sequence of practices aimed at providing timely emergency care, potentially leading to better patient outcomes. The embeddedness of practical wisdom has been subjectively interpreted and empirically studied in the healthcare literature [28, 32, 33], and this study expands the discourse while understanding the practical wisdom in the practices of healthcare volunteers and their role in emergency care.

Our study employed a purposively selected sample of CFRs, CFR leads, ambulance clinicians and patients and their relatives in order to explore the practices of CFRs. Even though our data collection methods and analysis were consistent with the principles of qualitative research [34] and data validation was obtained through patient and public consultation, there was still possibility that negative experiences and concerns about suboptimal patient care and survival can be constructed in CFR practices. Future research should focus on exploring these negative experiences of CFR practices. More research is needed to identify ethical and legal implications of CFR scheme implementation and how these influence the practices of both CFRs and ambulance clinicians, who are on the front lines of emergency and prehospital care.

Our study contributes to the discourse of how CFRs and CFR schemes were implemented to deliver emergency and prehospital care. This study sheds light on the sequential practices of CFRs that helped emergency patients get the right care at the right time within the community. A strength of our study is that it employed the theory of practice wisdom in the interpretation of empirical CFR practices [35]. Moreover, we collected data from six ambulance services regions, which covers most rural communities in England. The practice of debriefing of the emerging data and study findings with our PPI panel provided data validation, thus strengthening the methodological rigour.

## Conclusion

Our study of CFR practices highlights some important opportunities in the prehospital emergency care and its implications for both the provision of healthcare services and academic impact on the emergency care scholarship. We noted some important CFR practices, that replicate other findings on CFRs and CFR schemes, that CFRs played an important role in the ambulance services in the United Kingdom. The significant CFR practices were identification of patient signs and symptoms, information sharing, rapid emergency response, and engagement with ambulance clinicians. This set of practices positively enhanced the health system response to emergency care and shortened the ambulance service response time in rural areas, increasing the desirability of CFR schemes. Moreover, we noted that the use of practice wisdom to interpret CFR practices led us to comprehend how ‘doing the right thing at the right time’ embodied the patient-specific context in which CFRs provided emergency care. These practices could also shape community expectations regarding the future evolution of CFRs and CFR schemes.

## Supplementary Information

**Additional file 1: Table S1. **Consolidated criteria for reporting qualitative studies (COREQ): 32-item checklist.

## Data Availability

The datasets generated and/or analysed during the current study are not publicly available due to the guidelines of the Human Research Ethics Committee, University of Lincoln  but are available from the corresponding author on reasonable request.

## Abbreviations

CFR: Community first responder
CPR: Cardiopulmonary resuscitation
EMS: Emergency medical service
e-PRF: Electronic patient record form
NHS: National health service
PPI: Patient and public involvement
PRF: Patient record form
