# Supplementary material for: “It's like a swan, all nice and serene on top, and paddling like hell underneath”: community first responders’ practices in attending patients and contributions to rapid emergency response in rural England, United Kingdom—a qualitative interview study
Source: Scand J Trauma Resusc Emerg Med. 2023 Feb 13;31:7. doi: 10.1186/s13049-023-01071-3 (PMC9924885; doi:10.1186/s13049-023-01071-3)
Supplement: Supplementary file 1 — Additional file 1: Table S1. Consolidated criteria for reporting qualitative studies (COREQ): 32-item checklist. [file 13049_2023_1071_MOESM1_ESM.docx]

**Table S1: Consolidated criteria for reporting qualitative studies (COREQ): 32-item checklist**

Developed from: Tong A, Sainsbury P, Craig J. Consolidated criteria for reporting qualitative research (COREQ): a 32-item checklist for interviews and focus groups. *International Journal for Quality in Health Care*. 2007. Volume 19, Number 6: pp. 349 – 357

| **No. Item** | **Guide questions/description** | **Reported on Page #** |
| --- | --- | --- |
| **Domain 1: Research team and reﬂexivity** |  |  |
| *Personal Characteristics* |  |  |
| 1. Interviewer/facilitator | Which author/s conducted the interview or focus group? | 6 |
| 2. Credentials | What were the researcher’s credentials? E.g. PhD, MD | Title page: MSc, PhD, MSc |
| 3. Occupation | What was their occupation at the time of the study? | 7 |
| 4. Gender | Was the researcher male or female? | Not reported. The purpose of the interviews was to understand the perceptions and experiences of participants on roles of community first responders in care delivery. Therefore, the gender of the researcher is inconsequential. |
| 5. Experience and training | What experience or training did the researcher have? | Title page: MSc, PhD, MSc |
| *Relationship with participants* |  |  |
| 6. Relationship established | Was a relationship established prior to study commencement? | Yes, through contacting for recruitment of participants, arrangement of interview meetings and brief introduction prior to interviews |
| 7. Participant knowledge of the interviewer | What did the participants know about the researcher? e.g. personal goals, reasons for doing the research | Not reported. The researcher introduced himself to participants as researching in the role of community first responders. |
| 8. Interviewer characteristics | What characteristics were reported about the interviewer/facilitator? e.g. Bias, assumptions, reasons and interests in the research topic | Not reported. |
| **Domain 2: study design** |  |  |
| *Theoretical framework* |  |  |
| 9. Methodological orientation and Theory | What methodological orientation was stated to underpin the study? e.g. grounded theory, discourse analysis, ethnography, phenomenology, content analysis | 6 - 7 |
| *Participant selection* |  |  |
| 10. Sampling | How were participants selected? e.g. purposive, convenience, consecutive, snowball | 6 - 7 |
| 11. Method of approach | How were participants approached? e.g. face-to-face, telephone, mail, email | 6 |
| 12. Sample size | How many participants were in the study? | 5. (also see table 1) |
| 13. Non-participation | How many people refused to participate or dropped out? Reasons? | No dropouts |
| *Setting* |  |  |
| 14. Setting of data collection | Where was the data collected? e.g. home, clinic, workplace | 7  . |
| 15. Presence of non-participants | Was anyone else present besides the participants and researchers? | No |
| 16. Description of sample | What are the important characteristics of the sample? e.g. demographic data, date | 6: Table 1 |
| *Data collection* |  |  |
| 17. Interview guide | Were questions, prompts, guides provided by the authors? Was it pilot tested? | 7 |
| 18. Repeat interviews | Were repeat inter views carried out? If yes, how many? | No |
| 19. Audio/visual recording | Did the research use audio or visual recording to collect the data? | 7 |
| 20. Field notes | Were ﬁeld notes made during and/or after the interview or focus group? | No |
| 21. Duration | What was the duration of the interviews or focus group? | 7 |
| 22. Data saturation | Was data saturation discussed? | 7 |
| 23. Transcripts returned | Were transcripts returned to participants for comment and/or correction? | No |
| **Domain 3: analysis and ﬁndings** |  |  |
| *Data analysis* |  |  |
| 24. Number of data coders | How many data coders coded the data? | 7 |
| 25. Description of the coding tree | Did authors provide a description of the coding tree? | 7-8 |
| 26. Derivation of themes | Were themes identiﬁed in advance or derived from the data? | 8 |
| 27. Software | What software, if applicable, was used to manage the data? | 8 |
| 28. Participant checking | Did participants provide feedback on the ﬁndings? | No |
| *Reporting* |  |  |
| 29. Quotations presented | Were participant quotations presented to illustrate the themes/ﬁndings? Was each quotation identiﬁed? e.g. participant number | 9-16 |
| 30. Data and ﬁndings consistent | Was there consistency between the data presented and the ﬁndings? | 9-16 |
| 31. Clarity of major themes | Were major themes clearly presented in the ﬁndings? | 9-16 |
| 32. Clarity of minor themes | Is there a description of diverse cases or discussion of minor themes? | 9-16 |
